# Application of machine learning in the diagnosis of vestibular disease

**DOI:** 10.1038/s41598-022-24979-9

**Published:** 2022-12-02

**Authors:** Do Tram Anh, Hiromasa Takakura, Masatsugu Asai, Naoko Ueda, Hideo Shojaku

**Affiliations:** Department of Otorhinolaryngology, Head and Neck Surgery, Faculty of Medicine, Academic Assembly, University of Toyama, 2630 Sugitani, Toyama City, Toyama Prefecture 930-0194 Japan

**Keywords:** Machine learning, Physical examination, Neurological disorders

## Abstract

Machine learning is considered a potential aid to support human decision making in disease prediction. In this study, we determined the utility of various machine learning algorithms in classifying peripheral vestibular (PV) and non-PV diseases based on the results of equilibrium function tests. A total of 1009 patients who had undergone our standardized neuro-otological examinations were recruited. We applied five supervised machine learning algorithms (random forest, adaboost, gradient boosting, support vector machine, and logistic regression). After preprocessing the data, optimizing the hyperparameters using GridSearchCV, and performing a final evaluation on the test set using scikit-learn, we evaluated the predictive capability using various performance metrics, namely, accuracy, F1-score, area under the receiver operating characteristic curve, precision, recall, and Matthews correlation coefficient (MCC). All five machine learning algorithms yielded satisfactory results; the accuracy of the algorithms ranged from 76 to 79%, with the support vector machine classifier having the highest accuracy. In cases where the predictions of the five models were consistent, the accuracy of the PV diagnostic results was improved to 83%, whereas it increased to 85% for the non-PV diagnostic results. Future research should increase the number of patients and optimize the classification methods to obtain the highest diagnostic accuracy.

## Introduction

Dizziness and vertigo are symptoms that frequently induce patients to visit a physician. However, determining the cause of these symptoms is complicated because of the wide range of diseases with which they are associated. Peripheral vestibular (PV) system dysfunction is one of the most common etiologies of vertigo^1^, causing conditions such as benign paroxysmal positional vertigo, vestibular neuritis, and Meniere’s disease^2^. The diagnostic criteria for PV disease are mainly based on the patient’s history and a systematic clinical examination of the vestibular, oculomotor, and cerebellar systems according to the clinically oriented diagnostic criteria of the Bárány Society^3^.

In our department, various equilibrium examinations, including the caloric test and the optokinetic nystagmus test, have been used as diagnostic methods. In addition, imaging tests such as brain magnetic resonance imaging (MRI), brain computed tomography (CT), temporal bone CT, and cervical vascular sonography are performed as needed in almost all patients. Emergency patients with vertigo/dizziness are not included among the subjects who undergo the examinations mentioned above. Our equilibrium examinations are performed on outpatients who visit our department, including patients who are referred from other hospitals and patients who are admitted to our department due to vertigo/dizziness. In addition, we also examine some patients after admission to various other departments in our hospital, such as the emergency, neurology, and neurosurgery departments. These patients often have difficult medical conditions resulting from undiagnosed, ineffective treatment and long-term persistence of symptoms. Therefore, the accurate diagnosis of these patients and the differentiation of patients with PV disease from others are important tasks for us.

Machine learning (ML) has been developing rapidly in recent years and is being used in many aspects of medicine, especially radiology, robotic surgical systems, and disease diagnosis^4–6^. Since the 1980s, computer-based algorithms in medicine, called “expert systems”, have been used to simulate the steps and decision-making processes in the specific field of otolaryngology^7,8^. Recently, ML has been studied as a useful software method to assist medical decision making for vestibular dysfunction^9–13^. These studies show that ML is becoming a potential solution to help physicians most effectively access and use large amounts of information to make accurate diagnoses. However, the patients we must diagnose in our daily practice vary in time from disease onset and in their symptom severity. The most important capability that we desire an ML system to have is the ability to distinguish between PV disease and non-PV disease in patients who are difficult to diagnose. This result influences the choice of both treatment and the next test to be performed.

The purpose of the present study was to evaluate the ML models created with various learning algorithms for binary classification between PV disease and non-PV disease using the datasets generated from our equilibrium examinations. Furthermore, we devised a method to optimize the performance of the ML model.

## Materials and methods

This study was approved by the Ethics Committee of the Toyama University Hospital, Toyama Japan (Approval Number: R2019003). All methods were performed in accordance with the relevant guidelines and regulations. In this study, informed consent was obtained by publishing an opt-out document on the website, based on the Ethical Guidelines for Medical and Health Research Involving Human Subjects established by the Ministry of Health, Labour and Welfare of Japan.

### Subjects

The data of 1009 patients who underwent equilibrium examinations in our department (the Department of Otolaryngology, Head and Neck Surgery, University of Toyama) in the 10-year span from 2009 to 2019 were retrieved. The number of patients with PV was 497, and the number with non-PV disease was 512 (611 males and 398 females overall; mean age, 55.6 years). PV disease and non-PV disease were diagnosed according to the International Classification of Vestibular Disorders of the Bárány Society^14^ and the guidelines of the Japan Society for Equilibrium Research^15^ (Table 1). For example, small acoustic neuromas corresponding to Koos^16^ grade I or II were classified in the PV disease group. Patients who were confirmed to have unilateral PV dysfunction but could not be diagnosed as having an established clinical entity were classified in the PV disease group and considered to have inner ear disorder. Patients who were evaluated to have normal PV function and in whom central nervous system disease was ruled out by neurological examinations and brain MRI/magnetic resonance angiography (MRA) or brain CT were classified in the non-PV disease group and considered to have dizziness syndromes of unclear etiology. However, even if brain MRI/MRA and brain CT did not show any abnormalities, patients who showed normal vestibular function but showed abnormalities in the optokinetic nystagmus test and eye tracking test were classified in the non-PV disease group and considered to have central balance disorder. These patients often showed downbeat nystagmus, failure of fixation suppression, and abnormal eye movement. Although the cause of persistent postural-perceptual dizziness may be rooted in the PV system, these symptoms are thought to be modified by other factors. For this reason, patients with these symptoms were classified in the non-PV disease group.

**Table 1:** Demographic data and clinical diagnosis of patients.

Peripheral vestibular disease	n = 497
Age, mean in years (range)*	55.9 (7–93)
**Sex, n (%)**^**#**^	
Male	220 (44.3)
Female	277 (55.7)
**Diagnosis, n (%)**	
Acoustic tumor (Koos I/II)	18 (3.6)
Benign paroxysmal positioning vertigo	44 (8.9)
Bilateral vestibulopathy	2 (0.4)
Cholesteatoma/chronic otitis media	29 (5.8)
Delayed endolymphatic hydrops	19 (3.8)
Facial nerve paralysis/Hunt syndrome	13 (2.6)
Inner ear disorder	156 (31.4)
Meniere’s disease	120 (24.1)
Perilymphatic fistula	4 (0.8)
Sudden deafness with vertigo	44 (8.9)
Vestibular neuritis	48 (9.7)

Non-peripheral vestibular disease	n = 512
Age, mean in years (range)*	55.3 (7–91)
**Sex, n (%)**^**#**^	
Male	391 (76.4)
Female	121 (23.6)
**Diagnosis, n (%)**	
Brain infarction/bleeding	22 (4.3)
Brain tumor	20 (3.9)
Central balance disorder	87 (17.0)
Congenital nystagmus	8 (1.6)
Disembarkment syndrome	3 (0.6)
Dizziness syndromes of unclear etiology	32 (6.3)
Head injury	9 (1.8)
Hemodynamic orthostatic dizziness/vertigo	159 (31.1)
Migraine	7 (1.4)
Other central nervous system disease (n < 5)	31 (6.1)
Parkinsonism	5 (1.0)
Persistent postural-perceptual dizziness	23 (4.5)
Psychogenic dizziness	33 (6.4)
Spinocerebellar degeneration	24 (4.7)
Vertebrobasilar insufficiency/vertebral basilar artery stenosis	49 (9.6)

*Mann–Whitney *U* test, P = 0.727.

^#^Chi-squre test, P < 0.0001.

All patients underwent our standardized neuro-otological examinations, listed as number (No.) 1 to No. 16 in Table 2. These 16 examinations yielded a total of 44 features, which could be divided into two types: continuous and categorical. Continuous features with numerical values were used as they were, and categorical features were coded as integers from 0 to 3. Examinations No. 1 to No. 14 were performed as routine examinations, and examinations No. 15 and No. 16 were added as needed. In the caloric test (No. 6), we injected air currents at 24 °C and 50 °C (6 L/min) into each ear canal for 60 s with the patient’s eyes closed. The maximal slow-phase velocity (MSPV), canal paresis percentage (CP%), and directional preponderance percentage (DP%) of the caloric nystagmus were recorded after each irrigation, and the CP% and DP% were calculated from the MSPV according to Jonkees’ formula^17^. In our department, if the CP is ≥ 20%, the ear with the lower response is assumed to have unilateral vestibular hypofunction, indicating an abnormal caloric reflex. Bilateral vestibular hypofunction as evaluated by MSPV is defined as < 6°/s in each ear after caloric stimulation^18^. The failure of fixation suppression test (No. 7) started at 80 s after the beginning of the air current and continued for 10 s. The patient, with both eyes open, stared at an optotype^19,20^. The pendular sinusoidal rotation test (No. 8) was performed with rotation of the chair at 0.l Hz, amplitude 240°, maximum velocity of 75.4°/s, with the patient’s eyes closed^21^. In the eye tracking test (No. 9), the patient gazed at and pursued an optotype lamp (viewing angle 20 degrees, frequency 0.3 Hz) that moved left and right^22^. In the optokinetic nystagmus test (No. 10), 12 striations were projected onto a hemispherical drum. The striations began to rotate in the clockwise (CW) direction of 1°/s and accelerated until a velocity of 100°/s was reached. Next, the striations began to rotate in the counterclockwise (CCW) direction^23^. Two neuro-otology specialists (M.A. and H.S.) certified by the Japan Society for Equilibrium Research evaluated the waveforms from electronystagmography (ENG) by visual inspection and diagnosed all ENG findings. Stabilometry (No. 11) was performed according to the Japanese standard^24^. The Mann test (No. 12) was performed during tandem standing for 30 s with the eyes open and 30 s with the eyes closed, and then the positions of the front and back legs were reversed^25^. In the Fukuda stepping test (No. 13), the patient stood upright with eyes closed and arms extended forward and took 50 steps^26,27^. In the Schellong test (No. 14), blood pressure was measured twice in a recumbent position and 3 additional times: immediately after standing and 5 and 10 min later^28^. The galvanic body-sway test (GBST) (No. 15) evaluates the body-sway response induced by 0.2 mA and 0.4 mA electrical stimulation applied to the retroauricular area. Bipolar rectangular current stimulation lasting for 3 s was repeated 10 times, alternating between the left and right, as the patient stood on the stabilometer with his or her feet close together^29^. The stimulus conditions of the cervical vestibular evoked myogenic potential (cVEMP) test (No. 16) were a click sound of 0.1 ms, a frequency of 5 Hz, and a sound pressure level of 105 dB. Two hundred reaction waveforms were summed^30^.

**Table 2:** Equilibrium examinations and each feature’s name.

Examinations	Features (n = 44)	Formulas and criteria (Appendix 1)
1. Spontaneous nystagmus test	SpontanNy	1
2. Nystagmus during neck torsion to the right or left	NeckTor_R	
	NeckTor_L	
3. Nystagmus during neck compression to the right or left carotid sinus	NeckComp_R	
	NeckComp_L	
4. Positional nystagmus of 6 head positions	PositionalNy1-PositionalNy6	
	PositionalNum	2
5. Positioning nystagmus of 6 head positions	PositioningNy1-PositioningNy6	1
	PositioningNum	2
6. Bithermal caloric test	Caloric_CP%	3
	Caloric_DP%	4
	Caloric_CP	5
	Caloric_DP	6
7. Failure of fixation suppression test	FFS	7
8. Pendular sinusoidal rotation test	PSRT_R	8
	PSRT_L	
	PSRT_DP%	9
9. Eye tracking test	ETT	10
10. Optokinetic nystagmus test	OKN_CW	11
	OKN_CCW	
	OKN_DP%	12
11. Stabilometry	Envelop area_Op	13
	Envelop area_Cl	
	Sway Length_Op	14
	Sway Length_Cl	
	Romberg_Area	15
	Romberg_Length	
12. Mann test	Mann	16
13. Fukuda stepping test	Stepping	17
14. Schellong test	Schellong	18
15. Galvanic body sway test	GBST_R	19
	GBST_L	
16. Cervical vestibular evoked myogenic potentials	cVEMP_R	20
	cVEMP_L	

*Ny* nystagmus, *R* right, *L* left, *CP* canal paresis, *DP* directional preponderance, *MSPV* maximal slow phase velocity (degree/second), *FFS* failure of fixation suppression test, *PSRT* pendular sinusoidal rotation test, *ETT* eye tracking test, *OKN* optokinetic nystagmus, *CW or CCW* clockwise or counterclockwise, *Op* open, *Cl* close, *GBST* galvanic body sway test, *cVEMP* cervical vestibular evoked myogenic potentials.

### Steps in the machine learning classification method

In the present research, we used supervised ML to perform classification, which aims to predict the categories of new observations based on a training set of data whose categories are known^31^. The program was created on Google Colaboratory using Python version (v) 3.7.12, scikit-learn^32^ v1.0.2, NumPy v1.21.5, SciPy v1.4.1, Pandas v1.3.5, and Matplotlib v3.2.2. Five well-known algorithms, random forest (RF), adaboost (AB), gradient boosting (GB), support vector machine (SVM), and logistic regression (LR), were adopted. These algorithms have been used in a large number of treatises and specialized books based on an established theory^33–39^. The steps in classification are as follows.

#### Import the data

From the results of the 1009 patients, we created a CSV data file consisting of 44 features and target categories (PV = 0, non-PV = 1). After the CSV data were imported into the program, they were preprocessed to ensure the accuracy of future predictions^40^.

#### Split the data

**Figure 1:**
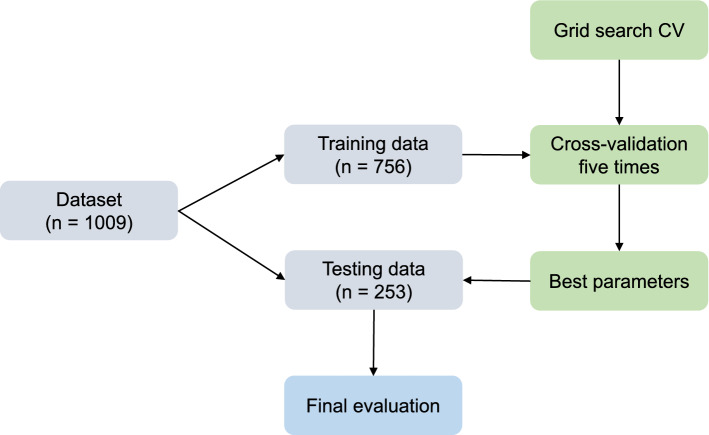
Overview of our machine learning process.

The preprocessed dataset was randomly divided into 75% training data (n = 756) and 25% testing data (n = 253), as shown in Fig. 1. The randomness of splitting for training and testing data was controlled via the "random_state" parameter in scikit-learn.

#### ML and predictions

ML was performed to create the best model using the training data. In the learning process, various parameters in the algorithm are automatically adjusted. However, some parameters need to be determined by a human to achieve the best prediction^41^. These variables, known as hyperparameters, can be set using GridsearchCV in scikit-learn^32^. By using GridsearchCV for each model, we could select the best hyperparameters and create the best model with each of the 5 algorithms. Thereafter, the best models were applied to the test data, as shown in Fig. 1, to create the final evaluation output. In the following description, the models obtained under the condition of random_state = 0 are presented as the best models. Furthermore, we performed 10 replicates of the whole process from splitting the data to applying the new best model for the test data by changing the random state and calculated average values such as accuracy.

### Test measures

In the binary classification, one of the two predicted groups was called the negative group (N), and the other was called the positive group (P). We defined PV disease as (N) and non-PV disease as (P). The confusion matrix is commonly used to evaluate the diagnostic ability of classifiers. In Table 3, the basic framework of the confusion matrix^32^ displays the number of predictions by each model in each of four categories: TP (true positive), FP (false positive), FN (false negative), and TN (true negative).

**Table 3:** Basic framework of the confusion matrix.

Confusion matrix	Predicted class	
	Negative (class 0)	Positive (class 1)
**Real class**		
Negative (class 0)	Number of true negatives	Number of false positives
Positive (class 1)	Number of false negatives	Number of true positives

The six test measures used for evaluating the predictive performance of ML are as follows: accuracy, precision, recall (also known as sensitivity), area under the receiver operating characteristic curve (AUC-ROC), F1-score, and Matthews correlation coefficient (MCC). The first five measures are displayed as numerical values ranging from 0 to 1, whereas MCC is displayed as numerical values from − 1 to 1. The greater each value is, the higher the predictive performance.

### Statistical analysis

The Mann‒Whitney *U* test was used for statistical evaluation of age, precision, recall, and F1-score between PV and non-PV. The Chi-square test was used for statistical evaluation of gender proportion. BellCurve for Excel v3.21 (Social Survey Research Information Co., Ltd., Japan) was used for the analysis, and P < 0.05 was considered statistically significant.

## Results

**Figure 2:**
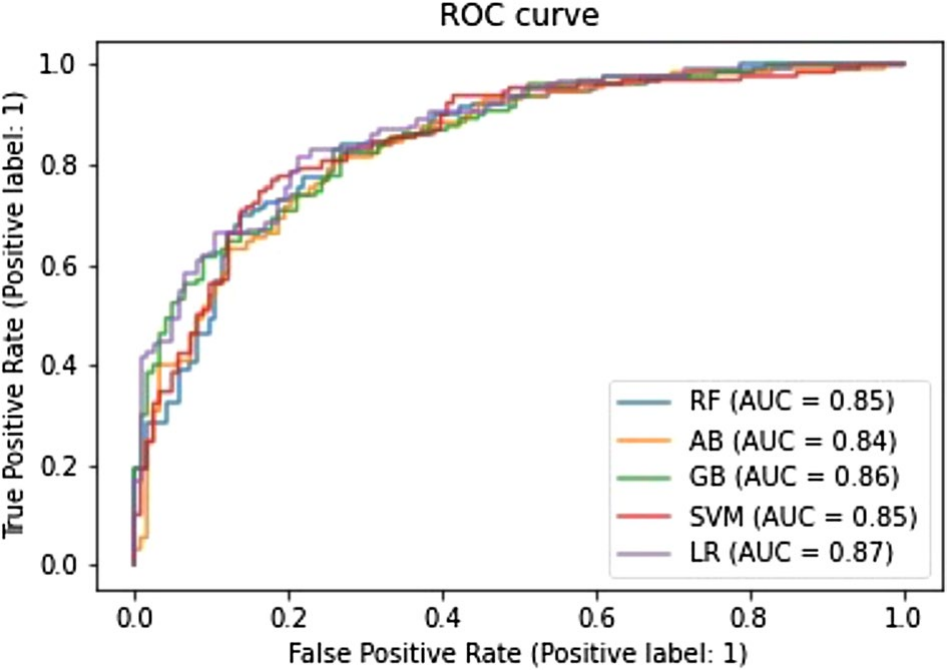
Comparison of ROC curves among the five machine learning models. *ROC* receiver operating characteristic, *AUC* area under the curve, *RF* Random Forest, *AB* AdaBoost, *GB* Gradient Boosting, *SVM* Support Vector Machine, *LR* Logistic Regression.

We created five models of classifiers for binary classification using a training set (n = 756) and applied them to a test set (n = 253), which was composed of PV (n = 123) and non-PV (n = 130) data. The predictive performance of the best models and the average data after ten different iterations are summarized for each of the five algorithms in Tables 4 and 5 with the six evaluation measures. Among the five models, the best single-trial performance metrics were as follows: 79% for SVM in accuracy, 0.87 for LR in AUC-ROC, and 0.57 for SVM in MCC (Table 4). By contrast, when the results were averaged across ten iterations, LR was the top performers on all three metrics, with accuracy of 77%, an AUC-ROC of 0.85, and an MCC of 0.54 (Table 5). Although LR and SVM showed better predictive performance than the other models, there was no remarkable difference among the five models. The AUC-ROC is one of the most commonly used metrics in evaluating the performance of binary classifiers. Based on a comparison of ROC curves among the five ML models, as shown in Fig. 2, all five algorithms achieved high AUC-ROC values from 0.85 to 0.87. All models were similar not only in their high AUC-ROC values but also in the shape of their ROC curves, indicating that all of the classifiers yielded consistently good results.

**Table 4:** The best performance of different machine learning algorithms.

ML classifiers	Accuracy	AUC-ROC	MCC	Precision		Recall		F1-score	
				PV	Non-PV	PV	Non-PV	PV	Non-PV
RF	0.77	0.85	0.55	0.76	0.79	0.78	0.77	0.77	0.78
AB	0.76	0.84	0.52	0.74	0.78	0.78	0.74	0.76	0.76
GB	0.76	0.86	0.52	0.76	0.76	0.74	**0.78**	0.75	0.77
SVM	**0.79**	0.85	**0.57**	**0.78**	**0.80**	0.79	**0.78**	**0.78**	**0.79**
LR	0.78	**0.87**	0.56	0.76	**0.80**	**0.80**	0.75	**0.78**	0.78
Average	0.77	0.85	0.54	0.76*	0.79*	0.78^ns^	0.76^ns^	0.77^ns^	0.78^ns^
SD	0.01	0.01	0.02	0.01	0.02	0.02	0.02	0.01	0.01

Bold letter indicates best score in each measure.

*ML* machine learning, *AUC-ROC* area under the receiver operating characteristic curve, *MCC* Matthews correlation coefficient, *PV* peripheral vestibular disease, *non-PV* non-peripheral vestibular disease, *RF* Random Forest, *AB* AdaBoost, *GB* Gradient Boosting, *SVM* Support Vector Machine, *LR* Logistic Regression, *SD* standard deviation. *P < 0.05 between PV and non-PV, *ns* no significant difference between PV and non-PV.

**Table 5:** The average performance of different machine learning algorithms after ten iterations.

ML classifiers	Accuracy	AUC-ROC	MCC	Precision		Recall		F1-score	
				PV	Non-PV	PV	Non-PV	PV	Non-PV
RF	0.74	0.83	0.49	0.76	0.73	0.70	0.78	0.73	0.75
AB	0.73	0.81	0.47	0.74	0.73	0.73	0.74	0.73	0.74
GB	0.75	0.83	0.49	0.76	0.73	0.71	0.78	0.73	0.75
SVM	0.75	0.84	0.51	0.76	0.75	**0.74**	0.76	0.75	0.76
LR	**0.77**	**0.85**	**0.54**	**0.78**	**0.76**	**0.75**	**0.79**	**0.76**	**0.77**
Average	0.75	0.83	0.50	0.76^ns^	0.74^ns^	0.73*	0.77*	0.74^ns^	0.75^ns^
SD	0.01	0.01	0.03	0.01	0.01	0.02	0.02	0.01	0.01

Bold letter indicates best score in each measure.

*ML* machine learning, *AUC-ROC* area under the receiver operating characteristic curve, *MCC* Matthews correlation coefficient, *PV* peripheral vestibular disease, *non-PV* non-peripheral vestibular disease, *RF* Random Forest, *AB* AdaBoost, *GB* Gradient Boosting, *SVM* Support Vector Machine, *LR* Logistic Regression, *SD* standard deviation. *P < 0.05 between PV and non-PV, *ns* no significant difference between PV and non-PV.

Table 4 also shows the best model results by diagnostic category. The best precision values were 0.78 by SVM for PV and 0.80 by SVM and LR for non-PV. The best recall values were 0.80 by LR for PV and 0.78 by GB and SVM for non-PV. The best F1-scores were 0.78 by SVM and LR for PV and 0.79 by SVM for non-PV. Apart from the individual model's superiority, the average precision of non-PV showed a higher value than that of PV, with 0.79 for non-PV and 0.76 for PV (P < 0.05). The average recall was 0.78 for PV and 0.76 for non-PV (no significant difference). The average F1-scores were 0.77 for PV and 0.78 for non-PV (no significant difference). In contrast, when the average results of each model were calculated after ten different iterations (Table 5), LR performed best on all metrics: precision (0.78 for PV and 0.76 for non-PV), recall (0.75 for PV and 0.79 for non-PV), and F1-score (0.76 for PV and 0.77 for non-PV). Although these values were slightly lower than the best model results, no significant difference was observed.

**Figure 3:**
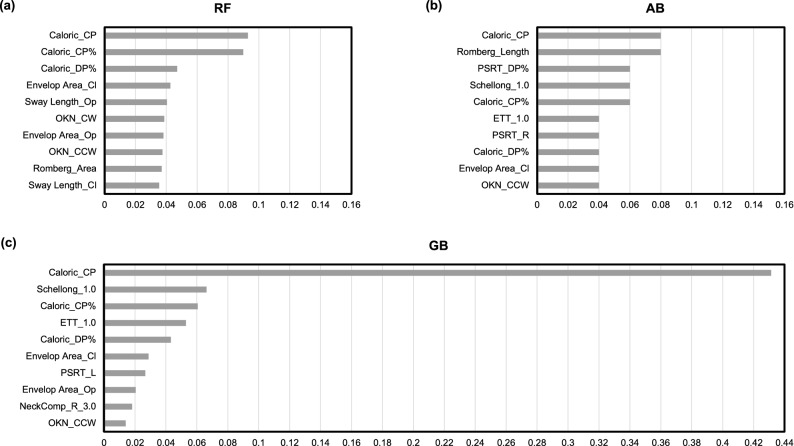
Top 10 most important features, ranked from high to low by classifier models. (**a**) Random forest. (**b**) Adaboost. (**c**) Gradient boosting. *RF* Random Forest, *AB* AdaBoost, *GB* Gradient Boosting, *CP* canal paresis in caloric test, *DP* directional preponderance in caloric test, *PSRT* pendular sinusoidal rotation test, *R* right, *L* left, *Op* open, *Cl* close, *OKN* optokinetic, *CW* clockwise, *CCW* counterclockwise, *FFS* failure of fixation suppression test, *ETT* eye tracking test.

An index showing how much each of the features contributed to the prediction of a given model can be calculated by the property of “feature_importances” in scikit-learn. The “feature_importances” ranking indicates which features may be most relevant or least relevant to the research objective. The RF method is the most common method in feature importance selection and rankings^42^. In our research, the feature importance of RF, AB, and GB was ranked based on the selected frequency of a variable as a decision node of decision trees. We used all of these classifiers to rank the importance of variables according to their discriminative performance. Out of the forty-four features, the importance of each of the top ten selected features is presented in rank order in Fig. 3. Each feature was scored with a numerical value ranging between 0 and 1, where 0 means “not used at all” and 1 means “perfectly predicts the target” the higher the value, the more important the variable was. Among the features for evaluating vestibular function, the features of the caloric test (Caloric_CP, Caloric_CP%) were ranked highest in all three models. This confirms that CP in the caloric test is a parameter that plays an important role in classifying PV versus non-PV disease. As for the features related to the stabilometry test, the Romberg ratio of sway length (Romberg_Length) was included in the top 10 features in the AB model, ranking as high as Caloric_CP. Other features, such as Envelop Area_Op, Envelop Area_Cl, Sway Length_Op, Sway Length_Cl, and Romberg_Area, were also present in the top rankings for the three models. Among the features for assessing cerebellar and brainstem function, two features of the optokinetic nystagmus test (OKN_CW, OKN_CCW) were included in the RF model. The features of the eye tracking test (ETT), Schellong test (Schellong), and pendular sinusoidal rotation test (PSRT_R, PSRT_L) were also present in the top rankings of the three models.

**Figure 4:**
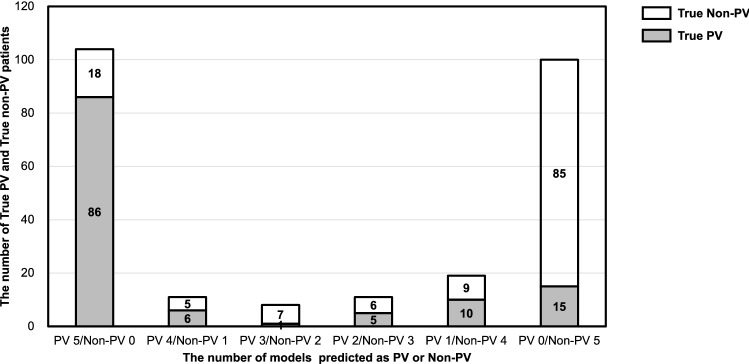
Evaluation of the accuracy of the five machine learning models in the testing dataset (n = 253). *PV* peripheral vestibular disease, *Non-PV* non-peripheral vestibular disease.

All five models in Table 4 were applied to evaluate the accuracy of PV versus non-PV classification in the 25% of data (253 cases) that formed the test set (Fig. 4). The numbers of models with matching predictions are shown in the six columns, which are labeled PV 0 to PV 5 and non-PV 0 to non-PV 5. In the graph, PV 5/non-PV 0 means that all five classifiers predicted PV and no model predicted non-PV. PV 0/non-PV 5 means that all five models predicted non-PV and no model predicted PV. The other labels indicate that the predictions were different depending on the model. In the first column, among the 104 patients predicted to have PV by all five models, 86 patients truly had PV and 18 patients had non-PV, which is equivalent to 83% accuracy. Similarly, in the last column, among the 100 patients predicted to have non-PV by all five models, 85 patients truly had non-PV and 15 patients had PV, which is equivalent to 85% accuracy. These percentages were higher than the accuracy of the models individually (Table 4).

## Discussion

In the present study, we evaluated the ability of ML models created from five algorithms to discriminate between PV disease and non-PV disease. These five algorithms were the commonly used RF, AB, GB, SVM, and LR methods and suggest the potential for supporting the prediction of vestibular disease diagnosis. Furthermore, our approach of combining all five ML classifier models was expected to support the prediction performance of each model individually.

All five models presented relatively good results by tuning the algorithms and choosing the best parameters using GridSearchCV. Among those, SVM in the five best models and LR in the average results seemed to be superior to those of the other models, with accuracy values of 79% and 77%, respectively. Joutsijoki et al.^43^ applied thirteen classification methods to oto-neurological disease classification and showed that the half-against-half (HAH) architecture with SVM achieved the best accuracy of 76.9% compared to the other classification methods, including LR, with an accuracy only slightly above 60%. Masankaran et al.^44^ used four classifier models (RF, SVM, k-nearest neighbor, and naïve Bayes) with the Dizziness Handicap Inventory questionnaire to distinguish benign paroxysmal positioning vertigo types with a best accuracy of 73.91%. Priesol et al.^11^ applied five classifier models (DT, RF, LR, AB, and SVM) and reported an overall accuracy of 76%. Compared to these reports, the performance of our best classifier had a higher accuracy of 79%. To further improve performance, we devised a method by combining all five models in the prediction data (Fig. 4). As a result, when the predictions of the five models matched in PV, the correct answer rate was 83%, and when they matched in non-PV, the correct answer rate was 85%. This result was superior to the accuracy of SVM and LR individually. However, when PV and non-PV predictions were presented simultaneously, the accuracies of SVM and LR were superior. Therefore, the combination of SVM and LR together with our new ML approach has the potential to diagnose PV disease and distinguish it from non-PV disease.

For otolaryngologists, it is important to reliably detect PV disease in patients with chaotic symptoms of vertigo/dizziness. However, the non-PV group included various diseases of cerebral etiology, such as brain tumor, brain infarction, spinocerebellar degeneration, vertebrobasilar insufficiency, and others for which a delayed diagnosis might lead to life-threatening consequences. Thus, ML should have a high predictive ability not only for PV diseases but also for non-PV diseases. This balance of predictive performance can be evaluated using precision, recall, and the F1-score. The F1-score is a measure that can comprehensively evaluate precision and recall. As shown in the best model results in Table 4, the precision average of the five models was better in non-PV than in PV. However, the F1-score averages of the five models were 0.77 for PV and 0.78 for non-PV. This result means that our models function well for both groups. Furthermore, the F1-scores of SVM were the best, with 0.78 for PV and 0.79 for non-PV. Thus, SVM appears to be a useful classifier for discriminating between the two disease groups.

Our dataset was established based on the clinical data of patients who were diagnosed by our 16 different types of equilibrium function tests, whereas previous studies usually used the most commonly performed vestibular tests, such as the caloric test and vestibulo-ocular reflex derived from the rotation test^11^, or used head impulse, gaze-evoked nystagmus, or a test of skew for differentiation of vestibular stroke and peripheral acute vestibular syndrome^9^. In Fig. 3, features related to the caloric test were the most important features, but the optokinetic nystagmus test, eye tracking test, Schellong test, pendular sinusoidal rotation test, and stabilometry also ranked in the top 10. Thus, the combination of multiple kinds of equilibrium examinations might help to increase the variety of features and improve the quality of the training dataset for ML. However, not all features in our dataset have equal importance. Determining which features yield the most predictive power is another crucial step in the model-building process.

This study has some important limitations, including the characteristics and size of the dataset and optimization of the models. In this study, ML was used to classify PV disease and non-PV disease, which include a wide range of diseases. Further studies using synthetic models in the classification of PV disease and a particular disease are needed to improve the diagnostic ability of ML. In addition, the number of study subjects was relatively modest, and other ML algorithms using advanced analytics techniques will be necessary to enhance the results. Moreover, the proportion of males and females has not been included in the training and test sets. Also, since we were unable to perform external validation, whether our ML model in the diagnosis of vestibular diseases is reproducible and generalizable has not been assessed. These could be subjects to be considered for future studies. Furthermore, obtaining extensive testing batteries as presented here will not tailor for clinical decision making in the setting of acutely dizzy patients in an emergency condition or in an outpatient center without examination equipment. Finally, even though ML can assist in making good predictions, it does not completely replace the physician. Especially with some diseases, which require patient‒physician interaction and critical thinking, the physician needs to make the final diagnosis.

## Conclusion

Diagnosis in neuro-otology is mainly deductive and based on the results of various vestibular function tests, which are difficult for otolaryngologists because of the experience requirements and the time-consuming nature of the tests. The current algorithm shows the effectiveness of using five ML models as an adjunct to distinguish between PV and non-PV diseases. The adoption of ML algorithms in clinical practice might free up physician time and enhance the accuracy and efficiency of the diagnosis and treatment of patients with PV disease.

## Supplementary Information

Supplementary Information.

## Data Availability

The datasets used and/or analyzed during the current study cannot be shared publicly so as to maintain the privacy of the individuals who participated in the study. The data will be shared on reasonable request to the corresponding author.
