# Supplementary material for: Application of machine learning in the diagnosis of vestibular disease
Source: Sci Rep. 2022 Dec 2;12:20805. doi: 10.1038/s41598-022-24979-9 (PMC9718758; doi:10.1038/s41598-022-24979-9)
Supplement: Supplementary file 1 — Supplementary Information. [file 41598_2022_24979_MOESM1_ESM.docx]

**Supplementary Information**

**Application of Machine Learning in the Diagnosis of Vestibular Disease**

Do Tram Anh, MD^†^, Hiromasa Takakura, MD, PhD^†^, Masatsugu Asai, MD, PhD*, Naoko Ueda, Hideo Shojaku, MD, PhD

Department of Otorhinolaryngology, Head and Neck Surgery, Faculty of Medicine, Academic Assembly, University of Toyama, Toyama, Japan

^†^Equally contributed as first authors to this work.

***Corresponding author:** Masatsugu Asai, Department of Otorhinolaryngology, Head and Neck Surgery, Faculty of Medicine, Academic Assembly, University of Toyama, 2630 Sugitani, Toyama City, Toyama Prefecture 930-0194, Japan

Tel.: +81764347368; Fax: +81764345038

E-mail address: [masai@med.u-toyama.ac.jp](mailto:masai@med.u-toyama.ac.jp)

**Supplementary information Appendix 1:** Feature types, formulas, and criteria of equilibrium examinations

1: categorical feature

0: No nystagmus

1: Nystagmus to the right

2: Nystagmus to the left

3: Other

2: continuous feature

Number of head positions nystagmus appeared (0 ~ 6)

3: continuous feature

Caloric_CP% = ((L24ºC・MSPV+L50ºC・MSPV) - (R24ºC・MSPV+R50ºC・MSPV)) * 100 / (L24ºC + R24ºC + L50ºC + R50ºC)

4: continuous feature

Caloric_DP% = ((L50ºC・MSPV+R24ºC・MSPV) - (R50ºC・MSPV+L24ºC・MSPV)) *100 / (L50ºC + R24ºC + L24ºC + R50ºC)

5: categorical feature

0: No CP

1: Suspected CP

2: Unilateral CP

3: Bilateral CP

6: categorical feature

0: No DP

1: Suspected DP

2: DP

7: categorical feature

0: Suppression ≥ 60% in 4 calorizations

1: Suppression < 60% in 1-2 calorizations

2: Suppression < 60% in 3-4 calorizations

8: continuous feature

PSRT_R = MSPV/75.4 in rotation to the right

PSRT_L = MSPV/75.4 in rotation to the left

75.4, rotation velocity of the chair

9: continuous feature

PSRT_DP% = (L・MSPV - R・MSPV) * 100 / (L・MSPV + R・MSPV)

10: categorical feature

0: Smooth

1: Saccadic

2: Ataxic

11: continuous feature

OKN_CW = MSPV in clockwise (CW) rotation

OKN_CCW = MSPV in counter-clockwise (CCW) rotation

12: continuous feature

OKN_DP% = (CW・MSPV - CCW・MSPV) * 100/ (CW・MSPV + CCW・MSPV)

13: continuous feature

Enveloped area (cm^2^) with eyes open and eyes closed

14: continuous feature

Sway length (mm) with eyes open and eyes closed

15: continuous feature

Romberg ratio of enveloped area and sway length

Romberg_Area = Area (eyes closed) / Area (eyes open)

Romberg_Length = Length (eyes closed) / Length (eyes open)

16: categorical feature

0: Standing ≥ 30 sec

1: Fall < 30 sec

17: categorical feature

0: RA < 45 deg and TA < 45 deg and TD < 50cm

1: RA ≥ 45 deg or TA ≥ 45 deg or TD ≥ 50cm

18: categorical feature

0: Decreased SBP < 20 mmHg and PP narrowing < 20 mmHg

1: Decreased SBP ≥ 20 mmHg or PP narrowing ≥ 20 mmHg

19: categorical feature

0: Normal response to 0.2 mA or 0.4 mA

1: Suspected

2: No response to 0.2 mA and 0.4 mA

20: categorical feature

0: 0.5 ≤ L/R ≤ 2.0

1: L/R < 0.5 or L/R > 2.0

**Abbreviations**: CP, canal paresis; R, right; L, left; MSPV, maximal slow phase velocity (°/s); DP, directional preponderance; PSRT, pendular sinusoidal rotation test; OKN, optokinetic nystagmus; CW or CCW, clockwise or counterclockwise; RA, rotation angle; TA, transition angle; TD, transition distance; SBP, systolic blood pressure; PP, pulse pressure; L/R, L/R ratio of amplitude.

**Supplementary information Appendix 2**: Pseudocode for machine learning

The csv file with the data of our 1009 patients has 52 columns which consists of 44 features, 1 target, and 7 additional information. The procedure from importing the classes of scikit-learn to creating the 5 best models is shown in PseudoCode. We avoided the use of scikit-learn terminology as much as possible and converted it into plain sentences. Subheadings and supplementary items are listed after “#”.

**PseudoCode**

**# Import functions of scikit-learn**

Import classes from scikit learn

# DecisionTreeClassifier, RandomForestClassifier, AdaBoostClassifier,

# GradientBoostingClassifier, SVC, LogisticRegression,

# GridSearchCV, train_test_split, StandardScaler, Confusion_matrix,

# Classification_report, RocCurveDisplay

**# Patients’ data**

Import CSV data file into Google Colaboratory Notebook

Create new data table used for ML from CSV file

**# Pre-Processing data**

If features columns have negative values

Create a new column for converting negative values to absolute values

Drop old columns with negative values

Drop columns of 7 additional information

Separate the target column from the data table

# Target column has 0 (peripheral) or 1 (non-peripheral)

Do one-hot-encoding for all categorical features

# Replace values with new feature columns with values of 0 and 1

Drop the first row

If columns have missing data

Create a new column that represent missing values with 1

Drop old columns before one-hot-encoding

If missing values exist in each column of continuous features

Calculate mean of each column

Fill missing values with the mean of each column

# **Split data**

Randomize the order of rows in a data table using “random_state”

# “random_state” fixes the order of random rows of data

# Default value of “random_state” is 0

If change of the randomization is needed

Change number of “random_state”

Split the data table into 75% training set and 25% test set

**# Random forest**

Create a list of hyperparameters to consider

Maximal depth of the tree is 4 to 20

Maximal number of estimators is 100, 150, 200

Divide training set into 5 subsets

# 4 subsets for training, 1 subset for validation

-------------------------------------------------------------------------------------

Compute GridSearchCV using RandomForestClassifier

# GridSearchCV is performed in line 55 to 61

Repeat training and validation for every combination of hyperparameters

Repeat training and validation changing subset 5 times

Average accuracy of 5 validations for every combination of hyperparameters

Find the best average value and the best combination of hyperparameters

Retrain the model with best hyperparameters using all training set

# This model is named as the best model

Print the best model

-------------------------------------------------------------------------------------

Do final evaluation with the best model using test set

# Evaluation is performed in line 65 to 79

Predict target class using the best model

# Peripheral vestibular class is expressed as 0

# Non-peripheral vestibular class is expressed as 1

Compute and Print Confusion matrix

# Number of TP, TN, FP, FN are displayed

Print results

Compute Classification_report

# Accuracy, precision, recall, f1-score for both classes is displayed

Print results

Compute Matthews correlation coefficient

Print result

Compute ROC Curve visualization and AUC

Print ROC graph and AUC

Compute feature importance

Print feature importance top10

**# Adaboost**

Create a list of hyperparameters to consider

Base model is DecisionTreeClassifier

Maximal depth of base model is 1 to 10

Weight applied in boosting is 0.5, 1.0, and 1.5

Maximal number of estimators is 50, 100, and 200

Divide training set into 5 subsets

# 4 subsets for training, 1 subset for validation

------------------------------------------------------------------------------------

Compute GridSearchCV using AdaBoostClassifier

Repeat line 55 to 61

------------------------------------------------------------------------------------

Do final evaluation with the best model using test set

Repeat line 65 to 79

**# Gradient boosting**

Create a list of hyperparameters to consider

Maximal depth of the tree is 1 to 5

Weight applied in boosting is 0.01, 0.05, 0.1, 0.15, and 0.2

Maximal number of estimators is 100,150, and 200

Divide training set into 5 subsets

# 4 subsets for training, 1 subset for validation

------------------------------------------------------------------------------------

Compute GridSearchCV using GradientBoostingClassifier

Repeat line 55 to 61

------------------------------------------------------------------------------------

Do final evaluation with the best model using test set

Repeat line 65 to 79

**# Support vector machine**

Create a list of hyperparameters to consider

First combination group

kernel is ‘rbf’

C is 0.001, 0.01, 0.1, 1, 10, and 100

Gamma is 0.001, 0.01, 0.1, 1, 10, and 100

Second combination group

Kernel is ‘linear’

C is 0.001, 0.01, 0.1, 1, 10, and 100

Divide training set into 5 subsets

# 4 subsets for training, 1 subset for validation

------------------------------------------------------------------------------------

Compute GridSearchCV using SVC

# GridSearchCV is performed in line 124 to 136

Standardize four training subsets

Subtract the average value from each feature value

Divide it by the standard deviation of each feature

Repeat training and validation for every combination of hyperparameters

Repeat line 124 to 127 changing validation subset 5 times

Average accuracy of 5 validations for every combination of hyperparameters

Find the best average value and combination of hyperparameters

Standardize all training sets

Subtract the average value from each feature value

Divide it by the standard deviation of each feature

Retrain the model with best parameter using all training set

# This model is named as the best model

Print the best model

------------------------------------------------------------------------------------

Do final evaluation with the best model using test set

# Evaluation is performed in line 140 to 155

Transform test set

# Use the formulas and numbers used for all training sets

# to transform the test set

Predict target class using the best model

# Peripheral vestibular class is expressed as 0

# Non-peripheral vestibular class is expressed as 1

Compute and Print Confusion matrix

# Number of TP, TN, FP, FN are displayed

Print results

Compute Classification_report

# Accuracy, precision, recall, f1-score for both classes is displayed

Print results

Compute Matthews correlation coefficient

Print result

Compute ROC Curve visualization and AUC

Print ROC graph and AUC

**# Logistic regression**

Create a list of hyperparameters to consider

First combination group

Solver is “liblinear”, “saga”

penalty is “l1”,” l2”

C is 0.1, 1, 10, and 100

Second combination group

Solver is “newton-cg”, “sag”, “lbfgs”

penalty is “l2”

C is 0.1, 1, 10, and 100

Divide training set into 5 subsets

# 4 subsets for training, 1 subset for validation

------------------------------------------------------------------------------------

Compute GridSearchCV using LogisticRegression

# GridSearchCV is performed in line 172 to 184

Standardize four training subsets

Subtract the average value from each feature value

Divide it by the standard deviation of each feature

Repeat training and validation for every combination of hyperparameters

Repeat line 172 to 175 changing validation subset 5 times

Average accuracy of 5 validations for every combination of hyperparameters

Find the best average value and combination of hyperparameters

Standardize all training sets

Subtract the average value from each feature value

Divide it by the standard deviation of each feature

Retrain the model with best parameter using all training set

# This model is named as best model

Print the best model

------------------------------------------------------------------------------------

Do final evaluation with the best model using test set

Repeat line 140 to 155 using best model for LogisticRegression
